# The association of household and child food insecurity with overweight/obesity in children and adolescents in an urban setting of Ethiopia

**DOI:** 10.1186/s12889-021-11392-6

**Published:** 2021-07-07

**Authors:** Sibhatu Biadgilign, Mekdes K. Gebremariam, Tennyson Mgutshini

**Affiliations:** 1 Department of Health Studies, College of Human Sciences, University of South Africa, Addis Ababa, Ethiopia; 2 Department of Nutrition, Faculty of Medicine, University of Oslo, P.O. Box 1046 Blindern, 0316 Oslo, Norway; 3 Department of Health Studies, College of Human Science, University of South Africa, PO Box 392, Preller Street, Pretoria, 0003 South Africa

**Keywords:** Food insecurity, Childhood, Adolescents, Overweight, Obesity, Ethiopia

## Abstract

**Background:**

Existing evidence on the association between food insecurity and childhood obesity is mixed. In addition, literature from developing countries in general and Ethiopia in particular on the nexus and impact of household and child food insecurity on childhood obesity in the context of urbanization remains limited. The objective of this study was to explore the association between household and child food insecurity and childhood obesity in an urban setting of Ethiopia.

**Methods:**

An observational population based cross-sectional study was conducted in five sub-cities of Addis Ababa. Multi-stage sampling techniques were employed to identify the study unit from the selected sub-cities. Multivariable logistic regression models with robust estimation of standard errors were utilized to determine the associations. Interactions by age and sex in the associations explored were tested.

**Results:**

A total of 632 children and adolescents-parent dyads were included in the study. About 29.4% of those in food secure households and 25% of those in food insecure households were overweight/obese. Similarly, 29.8% of food secure children and 22% of food insecure children were overweight/obese. Household and child food insecurity status were not significantly associated with child and adolescent overweight or obesity in the final adjusted models.

**Conclusions:**

Household and childhood food insecurity status were not associated with child and adolescent overweight/obesity in the study setting. Interventions aimed at combating overweight and obesity in the study setting should target children and adolescents irrespective of their food security status.

## Introduction

Food security is defined as access by all members at all times to enough food for a healthy and active life that provides a significant base for good health and nutrition [1]. Food security is mostly related to financial, economic and family issues that play dynamic roles in children’s nutritional status [1]. Food insecurity is a dynamic and complex issue, and not only can it lead to undernutrition and chronic hunger and long-term stunting, but also to overnutrition, which can lead to overweight and obesity. It can thus be related to a double burden of malnutrition [2].

Childhood overweight/obesity remains an important public health challenge globally due to the associated short and long-term health consequences [3–5]. Obesity-related cardiovascular disease in children is becoming more prevalent in conjunction with the rise in childhood obesity. Children with obesity are predisposed to an increased risk of cardiovascular morbidity such as dyslipidemia and insulin resistance and mortality during adulthood lifetime [6, 7]. There is evidence on the association of household and child food insecurity and childhood obesity [8, 9]. These associations could be attributed to a high consumption of poor quality high energy foods, more processed foods, and foods high in saturated fat, sugar, and salt which cause overweight and obesity [9–11].

The association between food insecurity and obesity among children is however far from consistent, as documented in systematic reviews of the literature [12–15]. Accordingly, positive, negative and null associations have been documented among children. The inconsistent associations documented among children make it difficult to draw any firm conclusions about the association between food security and weight status in this age group. There is thus a need for more evidence in this area. Since food insecurity and obesity are associated with common socioeconomic factors, it is important to control for these potential confounding variables when examining the association of food insecurity with childhood overweight status [16]. Against this background, the objective of this study was to determine the association of household and child food insecurity with childhood obesity in Ethiopia, by adjusting for important sociodemographic variables.

## Methods

### Sample and design

The study was an observational population based cross-sectional study conducted in representative samples of mother and child pairs in Addis Ababa city administration. The study was carried out in selected sub-cities in Addis Ababa, Ethiopia; namely, Bole, Gulele, Kolfe Keranio, Nifasilk Lafto and Yeka.

The source population was mother-child pairs at household level living in each sub-city during the study period. The study population was paired sampled school aged children with their mothers in the selected sub-cities. In this study, the following inclusion criteria were used to recruit participants: those children who are living with their mothers, those children who are in school aged (5 to 18 years old), mothers who can respond to the interviewer and school age children who lived in each of the sub-cities for at least 5 years. The exclusion criteria were: children who were permanently ill and the caregiver/mothers in a morbid state, severely ill (i.e. not able to provide the necessary information) and difficult to conduct or take any physical measurement (i.e. scoliosis and kyphotic deformities).

The sample size was calculated using single proportions sample size formula by using Epi Info statistical package (Centers for Disease Control and Prevention, Atlanta, U.S.A., 2010). The following parameters were used to calculate the sample size: proportion of children who were overweight in the population (P) is 9.5% [17], 95% CI(α = 0.05)[Z-The standard normal value at (100% − α) confidence level], d- 3% of Margin of error for sampling and 80%(β = 0.20) power. This gave a sample size of 367. So, by adding 15% for non-response rate and design effect of 1.5, total sample size was 634.. Multi-stage sampling technique were conducted to identify the study unit from selected sub-cites. From each sub-city, proportion to population sampling was applied to obtain the sample size. Simple random sampling method was applied to select districts and Kebeles (smallest administrative unit in government structure) in each sub-city. One child was selected from single-child households, and in some instance random selection of one child was done when the number of children in household more than one. In this case, a child was selected randomly using lottery method. In case of non-attendance of a qualified child in selected household, the next household was considered.

### Data collection instruments/tools

In this study, data were collected by a structured questionnaire originally developed in English. The content of the questionnaire included: socio-demographic characteristics (age and sex of child, age of mother, educational and occupational status of mother) and socio-economic indicators as well as household and child food security level. The tools were translated into Amharic and retranslated back to English by another expert to check and maintain its consistency. Data was collected by trained data collectors at home using standardized, structured and pre-tested tools.

### Data collection procedure

The quantitative study was conducted by interviewing mother-child dyads pair during data collection process. A team of data collectors with health professional background were recruited from the health facilities. Each team of interviewer was assigned in each selected sub-city, which consisted of one team supervisor, two females and two male interviewers. The supervisors oversaw the coordination aspect of data collection in sub-cities. One male and female interviewer was allocated per each household. Overall, 5 supervisors, 10 females and 10 male interviewers participated in the data collection process. Objective height and weight measurements were conducted. Weight measurements were obtained using lightweight, SECA mother-infant scales with a digital screen designed and manufactured under the guidance of The United Nations Children’s Fund (UNICEF). Height measurements were carried out using a measuring board in standing position. Weight and height of each child were measured after calibrating to the nearest 0.1 kg and 0.1 cm. In this study, height and weight measurements of children were converted into Z-scores based on WHO reference population considering their age and sex.

### Measurement

#### Outcome variable

The outcome variable used for analysis in this study was childhood overweight/obesity as a binary variable and defined as more than 1 SD above the median based on WHO growth reference [18].

### Independent variables

#### Household food security status

Household food security was measured using the Household Food Insecurity Access Scale of the Food and Nutrition Technical Assistance Project (HFIAS/FANTA)/US Agency for International Development (USAID), which offers information on behaviour and insights linked to household food insecurity status – anxiety and depression, inadequate diet quality and insufficient food intake or reducing quantity of food consumed. The HFIAS is a continuous measure of the degree of food insecurity mostly related to access in the household. The 10 questions/items which assess the dietary status were asked for a 30-days period preceding the survey. The households were categorized into three groups: food secure, mildly and moderately food insecure and severely food insecure [19].

#### Child food security status

Children’s Food Security status was measured by 8 items in the Children’s Food Security Scale survey module. The module developed by adjusting questions from the household food security survey module for direct administration to children. If the response to the affirmative to question with a row score of ≥2 then it is categorized as child food insecure otherwise food secure [20].

#### Other covariates/confounders

Because numerous demographic and socioeconomic characteristics of child and parent are often related to children’s overweight/obesity status and can also be related to food security status, a priori defined potential confounders based on biological and statistical considerations, were included. These variables were: socio-economic status/wealth index(poorest, poorer, middle, richer and richest), household asset index, age group of the children and adolescents in year (5–9, 10–14 and 15–18); sex of children and adolescents (male and female); sex of household head (male and female); age group of the household head in years (< 40 and ≥ 40); maternal education (no-formal education (are those who are Illiterate) and formal education (those who were literate)); maternal occupation (unemployed, private business and employed); marital status of the mother (married, divorced, widowed and separated); household size (numbers) (< 5 and ≥ 5) and type of school the child attends (private and public).

### Statistical analysis

The age- and sex-specific body mass index z-scores (BMIZ) among children and adolescents were calculated using the World Health Organization (WHO) 2007 reference data. In this study, descriptive analyses were used to characterize the variables under investigation. Chi-square test was used to explore the association between overweight/obesity and child and maternal characteristics. Explanatory variables that showed an association at *p* <  0.2 in bivariate analysis were included in the final models. Multivariable logistic regression model with robust estimation of standard errors, accounting for the clustering at the level of sub-city, were fitted to determine associations. Interaction of age and sex in the associations explored was checked. Statistical significance was defined as *P* <  0.05. Data was entered by SPSS Version 21 and analysis carried out by Stata 15.0 (Stata Corporation, College Station, TX) and WHO Anthro Plus software v1.02 (WHO, Geneva, Switzerland).

This study was conducted in accordance with the Declaration of Helsinki and all procedures involving human subjects were approved by the Institutional Review Boards of Departmental Higher Degrees Committee of the Department of Health Studies University of South Africa Ethical Clearance Committee for Research on Human Subjects (HSHDC/ 575/2016) and Addis Ababa City Administration Health Bureau (A/A/H/B/3542/227). Also, support letter was written from the University of South Africa Addis Ababa Regional Office to Addis Ababa City Administration Health Bureau. Official letters of co-operation from the above organizations were given to the respective sub-city and district administrator. Verbal informed consent was approved by the ethics committee of Departmental Higher Degrees Committee of the Department of Health Studies University of South Africa Ethical Clearance Committee for Research on Human Subjects and Addis Ababa City Administration Health Bureau. As well, verbal Informed consent was also obtained from each participant and confidentiality was assured. Additionally, for those children under the age of 18 years, verbal informed consent was obtained from their parents or caregivers. Assent was obtained from each participant.

## Results

A total of 632 children and adolescents-parent dyads were included in the study. About 76.1% of the respondents lived in a household with a male head. Regarding educational status of mother, 61.5% attended formal education. About 33.4% mothers were unemployed. Concerning marital status, 84.2% of mothers were married. The mean (SD) of the household size of participants was 5 (1.4) members (Table 1). About 48.4% of children and adolescents were male and around 19.8, 51.1 and 29.1% of the children and adolescents were within the age group of 5–9 years (middle childhood), 10–14 years (early adolescence) and 15–18 years (late adolescence). The mean (SD) of children’s age was 12.5 (± 2.96) years. About 53.5% of children and adolescents attended private school and the remaining attended public school (Table 1).

**Table 1:** Characteristics of participants according to overweight/ obesity status

Characteristics/variables	Categories	Childhood overweight /obesity		***P*** value
		Yes n (%)	No n (%)	
Age group of the children and adolescents, years	5–9	46 (25.27)	79 (17.56)	0.006
	10–14	98 (53.85)	225 (50.00)	
	15–18	38 (20.88)	146 (32.44)	
Sex of children and adolescents	Male	85 (46.7)	221 (49.1)	0.583
	Female	97 (53.3)	229 (50.9)	
Sex of household head	Male	140 (76.9)	341 (75.8)	0.760
	Female	42 (23.1)	109 (24.2)	
Age group of household head	< 40 years	22 (30.2)	176 (39.1)	0.036
	≥ 40 years	127 (69.8)	274 (60.9)	
Maternal education	No-formal education	63 (34.6)	180 (40)	0.208
	Formal education	119 (65.4)	270 (60)	
Maternal occupation	Unemployed	50 (27.5)	161 (35.8)	0.011
	Private business	30 (16.5)	96 (21.3)	
	Employed	102 (56.0)	193 (42.9)	
Marital status of the respondents	Married	153 (84.1)	379 (84.2)	0.747
	Divorced	17 (9.34)	33 (7.3)	
	Widowed	10 (5.49)	32 (7.11)	
	Separated	2 (1.10)	6 (1.33)	
Household size	< 5 Members	90 (49.5)	230 (51.2)	0.686
	≥5 Members	92 (50.5)	219 (48.8)	
Type of school where the child attend	Private	98 (53.8)	240 (53.3)	0.907
	Public	84 (46.2)	210 (46.7)	
Wealth index	Poorest	28 (15.56)	98 (22.07)	< 0.001
	Poorer	32 (17.78)	92 (20.72)	
	Middle	35 (19.44)	117 (26.35)	
	Richer	31 (17.22)	67 (15.09)	
	Richest	54 (30.00)	70 (15.77)	
Household food insecurity status	Food secure	151 (83.89)	363 (80.85)	0.332
	Mildly and moderately food insecure	17 (9.44)	39 (8.69)	
	Severely food insecure	12 (6.67)	47 (10.47)	
Child food insecurity status	Food secure	165 (90.66)	389 (86.64)	0.162
	Food insecure	17 (9.34)	60 (13.36)	

Employed*-Government and NGO employee

From the sample, 18.3% of households experienced food insecurity while 81.7% of households remained food secure. Regarding child food insecurity, about 87.8% were food secure children and adolescents. Overall, the prevalence of overweight/obesity among the sample based on WHO reference was 28.8% (95% CI: 25.29, 32.50).

About 29.4% of those in food secure households and 25% of those in food insecure households were overweight/obese. Similarly, 29.8% of food secure children and adolescents and 22% of food insecure children and adolescents were overweight/obese. There was no direct association between household food insecurity and overweight/obesity for children and adolescents of any age in bivariate analysis. However, child food insecurity status was significantly associated with overweight/obesity in bivariate analysis. Household and child food insecurity status did not have any association with child and adolescents’ overweight/obesity in the final model. Children and adolescents’ overweight/obesity was significantly associated with demographic and socio-economic variables (age group of children and adolescents, maternal occupation, wealth index, age group of household head) (Tables 2 and 3). We checked for interaction by age and sex in the association between household food insecurity and overweight/obesity and no interaction effect was observed in the final adjusted models.

**Table 2:** Association between overweight/obesity among children and adolescents and household food insecurity status

Characteristics/variables	Categories	Crude odd ratio	***P***-value	Adjusted odd ratio	P-value
Age group of the children and adolescents, years	5–9	1		1	
	10–14	0.75 (0.48–1.15)	0.190	0.52 (0.32–0.86)	0.011
	15–18	0.45 (0.27–0.74)	0.002	0.24 (0.13–0.44)	0.000
Maternal occupation	Unemployed	1		1	
	Private business	1.00 (0.60–1.69)	0.981	1.00 (0.56–1.80)	0.984
	Employed*	1.70 (1.14–2.53)	0.009	1.80 (1.13–2.87)	0.022
Wealth index	Poorest	1		1	
	Poorer	1.47 (0.80–2.69)	0.209	1.38 (0.73–2.60)	0.316
	Middle	1.08 (0.58–2.00)	0.812	1.05 (0.55–2.02)	0.881
	Richer	1.62 (0.88–2.97)	0.118	1.62 (0.81–3.17)	0.161
	Richest	3.14 (1.75–5.64)	0.000	3.23 (1.67–6.25)	0.001
Age group of household head	< 40 years	1		1	
	≥ 40 years	1.48 (1.03–2.14)	0.036	1.64 (1.06–2.56)	0.027
Maternal education	No-formal education	1		1	
	Formal education	1.26 (0.88–1.80)	0.208	1.09 (0.71–1.69)	0.689
Household food insecurity status	Food secure	1		1	
	Mildly and moderately food insecure	1.05 (0.57–1.91)	0.879	1.36 (0.69–2.71)	0.374
	Severely food insecure	0.61 (0.32–1.19)	0.148	0.65 (0.30–1.39)	0.269

Employed*-Government and NGO employee

**Table 3:** Association between overweight/obesity among children and adolescents and child food insecurity status

Characteristics/variables	Categories	Crude odd ratio	P-value	Adjusted odd ratio	P-value
Age group of the children and adolescents, years	5–9	1		1	
	10–14	0.75 (0.48–1.15)	0.190	0.52 (0.32–0.86)	0.010
	15–18	0.45 (0.27–0.74)	0.002	0.24 (0.13–0.44)	0.000
Maternal occupation	Unemployed	1		1	
	Private business	1.00 (0.60–1.69)	0.981	0.99 (0.55–1.76)	0.967
	Employed*	1.70 (1.14–2.53)	0.009	1.76 (1.11–2.80)	0.016
Wealth index	Poorest	1		1	
	Poorer	1.47 (0.80–2.69)	0.209	1.38 (0.73–2.59)	0.318
	Middle	1.08 (0.58–2.00)	0.812	1.05 (0.54–2.01)	0.892
	Richer	1.62 (0.88–2.97)	0.118	1.60 (0.82–3.12)	0.172
	Richest	3.14 (1.75–5.64)	0.000	3.18 (1.66–6.10)	0.000
Age group of household head	< 40 years	1		1	
	≥ 40 years	1.48 (1.03–2.14)	0.036	1.62 (1.04–2.50)	0.032
Maternal education	No-formal education	1		1	
	Formal education	1.26 (0.88–1.80)	0.208	1.08 (0.70–1.67)	0.718
Child food insecurity status	Food secure	1		1	
	Food insecure	0.67 (0.38–1.18)	0.164	0.78 (0.40–1.52)	0.470

Employed*-Government and NGO employee

## Discussion

The aim of this study was to determine the association of household and child food insecurity with child and adolescent overweight/obesity in an urban setting of Ethiopia. Results indicated that there was no association between household and child-specific food insecurity and childhood overweight/ obesity in the study setting.

Studies examining the relationship between household and child-specific food insecurity and childhood overweight/ obesity in developing countries are scarce. Studies in developed country settings however documented results of household food insecurity and childhood overweight/ obesity, which is in line with our findings [21–29], also for child-specific food insecurity [16, 21, 24, 27, 30–32]. One of these studies including children of different age groups showed that child-level food insecurity was not associated with obesity among 2 to 5 year-olds or 6 to 11 year-olds [32].

Different studies however showed different associations between food insecurity and overweight/obesity. In a recent study in the United States by Kral et al., the odds of a child being obese were found to be five times higher for children from food-insecure households compared with children from food-secure households. Furthermore, in the study, the majority of participating children from food-secure households consumed 3 to 4 snacks per day whereas a higher proportion of children from food-insecure households consumed 5 or more snacks per day [33]. Other studies also indicated that children living in food-insecure households are more likely to be obese compared to children who are food secure which support for the positive association between food insecurity and obesity [34–36]. Other studies on the other hand suggested that children living in food-insecure households were less likely to be obese [8, 37–39]. A systematic review conducted by Mary E. Morales (2016) highlights these inconsistencies in the findings of studies exploring the relationship between food insecurity and obesity are mixed [15].

The occurrence of food insecurity with overweight and obesity is still not fully understood. These inconsistencies could be explained by the fact that food insecurity may be obesogenic due to its relationship with unhealthy dietary patterns but could also lead to weight loss especially if it results in undernutrition [15]. According to Dhurandharm (2016), food-insecurity obesity nexus can be looked at with the notion of resource scarcity hypothesis, suggesting that low food security is associated with obesity because of high calorie, palatable foods consumed by low food-secure populations [40]. Furthermore, low food security can be associated with obesity because of the limited knowledge, time and resources that populations with low food security experience in terms of healthy eating and physical activity level [40–42].

Apart from this, other explanations in the literature demonstrated that the level of food insecurity is related to physical food environments that promote obesity. When compared to food-secure participants, marginal or low/very low food-secure mothers revealed that they use significantly more obesity-promoting foods at home, including microwavable or quick-cook frozen foods. Greater access to less healthful foods in food insecure households’ kitchen may be linked to poor diet quality and overweight /obesity of children in these households [43].

In addition to this, the mixed findings related to the association between food insecurity and overweight/obesity can also partly be due to differences in study designs. Furthermore, there are differences in the measurement of food insecurity between different studies. The current study includes all the eight questions for child specific food insecurity scale and ten full household food insecurity questions out of 18-item household food security survey module (HFSSM). A similar approach was used in research conducted by Bronte-Tinkew et al. and Casey [16, 44]. Other studies use few of the questions (subscale) from the scale [34, 45].

We tested for interactions by gender and age in the association between food insecurity and OW/OB and no significant interactions were detected in our study. Other studies have reported such interaction effects. A study from Brazil showed that the relationship between HFI and obesity was not significant for children, but among female adolescents, the likelihood of excess weight was 1.96 times higher among those with severe HFI when compared to their food-secure counterparts [46]. In a sample of U.S. children, no significant differences were found between household food security and BMI growth in males, whereas an association was detected among female children [47]. The mechanisms behind such differences between studies, including the role of contextual factors, should be explored further.

The study has strengths and limitations. Unlike other previous work where food insecurity is measured at the household level only, the current study also includes food insecurity of children and adolescents. In addition, we have included many covariates. A broad age group of participants, from school age to late adolescence, in a large urban center, was included. The study has some limitations. The associations between food insecurity and childhood obesity might differ between rural and urban settings, so the results cannot be generalized to rural settings. The cross-sectional nature of the study design used does not allow for inferences about causality between food insecurity status and childhood overweight / obesity. The socio-demographic composition of the sample also suggests an overrepresentation of families with higher affluence. This could at least partly explain the high prevalence of overweight/obesity documented in this study compared to other studies from Ethiopia, including in Addis Ababa [48]. This should be taken into consideration when interpreting the findings of this study. More work is warranted to confirm these findings with a larger nationally representative sample. Given the relatively small number of food insecure households and children/adolescents in the sample, future investigation with a larger sample size is warranted to confirm the findings.

## Conclusion

Household and childhood food insecurity status were not associated with childhood overweight/obesity in the study setting. Interventions aimed at combating overweight/obesity in the study setting should target children and adolescents irrespective of their food security status by make sure the target children have regular access to nutrient-rich (or nutrient-dense) foods.

## Data Availability

The data analyzed during this study are included in this article and are available from the corresponding author upon reasonable request.
